# Laparoscopic versus Open Emergency Surgery for Right Colon Cancers

**DOI:** 10.3390/diagnostics14040407

**Published:** 2024-02-13

**Authors:** Mohammad Iqbal Hussain, Guglielmo Niccolò Piozzi, Najmu Sakib, Rauand Duhoky, Filippo Carannante, Jim S. Khan

**Affiliations:** 1 Department of Colorectal Surgery, Portsmouth Hospitals University NHS Trust, Portsmouth PO6 3LY, UK; mihussain.cal@gmail.com (M.I.H.); guglielmopiozzi@gmail.com (G.N.P.);; 2 University of Portsmouth, Portsmouth PO1 2UP, UK; 3 Colorectal Surgery Unit, Fondazione Policlinico Universitario Campus Bio-Medico, 00128 Rome, Italy

**Keywords:** colon cancer, emergency surgery, minimally invasive surgery, laparoscopy, open approach

## Abstract

Background: A laparoscopic approach to right colectomies for emergency right colon cancers is under investigation. This study compares perioperative and oncological long-term outcomes of right colon cancers undergoing laparoscopic or open emergency resections and identifies risk factors for survival. Methods: Patients were identified from a prospectively maintained institutional database between 2009 and 2019. Demographics, clinicopathological features, recurrence, and survival were investigated. Cox regression analysis was performed for risk factor analysis. Results: A total of 202 right colectomies (114 open and 88 laparoscopic) were included. ASA III–IV was higher in the open group. The conversion rate was 14.8%. Laparoscopic surgery was significantly longer (156 vs. 203 min, *p* < 0.001); pTNM staging did not differ. Laparoscopy was associated with higher lymph node yield, and showed better resection clearance (R0, 78.9 vs. 87.5%, *p* = 0.049) and shorter postoperative stay (12.5 vs. 8.0 days, *p* < 0.001). Complication rates and grade were similar. The median length of follow-up was significantly higher in the laparoscopic group (20.5 vs. 33.5 months, *p* < 0.001). Recurrences were similar (34.2 vs. 36.4%). Open surgery had lower five-year overall survival (OS, 27.1 vs. 51.7%, *p* = 0.001). Five-year disease-free survival was similar (DFS, 55.8 vs. 56.5%). Surgical approach, pN, pM, retrieved LNs, R stage, and complication severity were risk factors for OS upon multivariate analysis. Pathological N stage and R stage were risk factors for DFS upon multivariate analysis. Conclusions: A laparoscopic approach to right colon cancers in an emergency setting is safe in terms of perioperative and long-term oncological outcomes. Randomized control trials are required to further investigate these results.

## 1. Introduction 

Colon cancer is the third most common cancer worldwide [1]. It presents as an emergency in 30% of cases [2], with large bowel obstructions accounting for 80% of emergencies and perforation for 20% [3,4]. Emergency colorectal cancer patients are less often treated with curative intent [5] and usually have poorer survival [6,7,8]. Female sex [2] and older age present higher risk of emergency presentation [9].

Right colectomy with primary anastomosis is the preferred option for obstructive right colon cancers, while end ileostomy with colonic fistula is considered a valid alternative if primary anastomosis is unsafe or the patient is unstable [3]. The open approach has been the preferred approach [10]. However, emergency laparotomies are associated with significant morbidity (40–60%) and mortality rates (3–11%) [11].

Laparoscopy provides significantly better postoperative outcomes (less blood loss, less pain, earlier gastrointestinal recovery, and fewer wound complications), with similar oncological outcomes to the open approach in the elective setting; therefore, it is generally considered the gold standard [12,13]. However, the role of laparoscopy in emergency resections for emergency colon cancers remains unclear [14]. 

Adoption of laparoscopy for emergency cases is challenging due to patient (high-risk for hemodynamic instability and/or sepsis), surgeon (availability of laparoscopically experienced teams out of hours), and technical (reduced operative space following dilated and obstructed small bowel) complexities [15].

Few studies show that laparoscopy is feasible and safe as a primary approach in the emergency setting [15,16]; however, studies reporting long-term oncological outcomes are missing.

This study compares perioperative and oncological long-term outcomes of right colon cancers undergoing laparoscopic or open approaches in the emergency setting and identifies risk factors for oncological survival.

## 2. Method

### 2.1. Study Population

This retrospective study evaluated a consecutive series of emergency right colon resections performed with laparoscopic or open approaches between January 2009 and December 2019. Data were extracted from a prospectively maintained colorectal and National Emergency Laparotomy Audit (NELA) database in a tertiary referral centre. The Institutional Review Board approved the study (IRAS ID 293129). All patients provided informed consent for research studies.

The primary endpoint was to compare perioperative and oncological long-term outcomes of right colon cancers undergoing laparoscopic or open approaches in the emergency setting.

The secondary endpoint was to identify risk factors for oncological survival.

**Figure 1:**
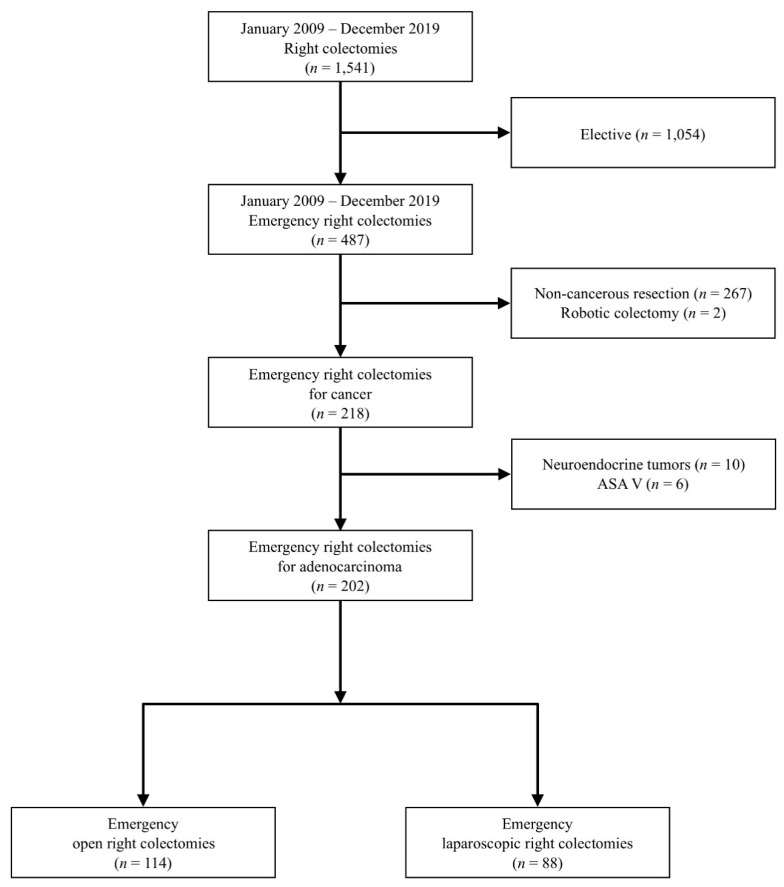
Patient selection flowchart.

Inclusion criteria (Figure 1): (1) right colon cancer (from ileocecal valve to mid-transverse colon proximal to the splenic flexure); (2) adenocarcinoma on postoperative histopathology; (3) emergency surgery; (4) open and laparoscopic approaches; (5) age ≥18 years. Exclusion criteria: (1) elective surgery; (2) other combined colorectal resections; (3) ASA V; (4) robotic approach.

Resections performed within 24–48 h of presentation with acute abdomen was defined as emergency resection. Participating surgeons were experienced colorectal surgeons who were proficient in managing both open and laparoscopic colorectal resections in the emergency setting. The decision to operate by laparoscopic or open approach rested on the on-call surgeon’s preference and expertise, taking into consideration the patient’s physiological status.

Diagnosis was confirmed using thoracic/abdominopelvic computed tomography. All patients were operated on according to a designated emergency list. No patients received bowel preparation. Thromboprophylaxis and prophylactic antibiotics were used routinely at induction.

### 2.2. Surgical Technique

All patients underwent emergency surgery after fluid deficits and electrolyte imbalances had been corrected. All surgeries were performed with open or laparoscopic approaches according to each surgeon’s choice and expertise, clinical condition, and platform/surgical team availability. The laparoscopic approach was chosen after careful clinical assessment with the anaesthetist.

Open right hemicolectomy was performed by midline laparotomy. Thorough abdominal inspection was performed. The ileocolic vascular pedicle, the right colic (if present), and the right branch of the middle colic artery were identified, ligated, and divided. The distal ileum (>15 cm from the ileocaecal junction) and the transverse colon were divided with a GIA^TM^ stapler (Medtronic, Minneapolis, MN, USA). An antiperistaltic side-to-side ileocolic anastomosis was accomplished with the GIA^TM^ stapler. Post-operative antibiotics were given if clinically indicated.

The laparoscopic approach was performed with four ports (12 mm at umbilicus, 5 mm suprapubic, 12 mm left lower quadrant, 5 mm left upper quadrant). After a thorough inspection of the liver, large and small bowel, and peritoneal surfaces the right colon was mobilised with a medial-to-lateral approach. Once the ileocolic pedicle was isolated, it was divided using Hem-o-lok (Teleflex, Wayne, PA, USA) or vascular staplers. The right colic and middle colic vessels were identified and divided similarly. Once the mobilisation was completed, the pneumoperitoneum was interrupted. The umbilical port was extended vertically, and a wound protector was applied. The mobilised right colon was delivered and divided in between occlusion clamps. Anastomosis was accomplished extracorporeally. Conversion was defined as the unintended extension of the midline incision to a laparotomy.

### 2.3. Pathological Report

During data review, the pathological staging was modified according to the American Joint Committee on Cancer (AJCC) 8th edition staging system [17].

### 2.4. Postoperative Outcomes and Follow-up

Enhanced recovery programmes were the standard of care post-operatively for all patients.

Postoperative complications, defined as adverse events occurring within 30 days from surgery, were assessed through the Clavien–Dindo classification [18]. Thirty-day mortality was recorded.

Adjuvant chemotherapy was offered following multidisciplinary team discussion.

Institutional postoperative follow-up protocol was physical examination every three months for two years; annual thoracic/abdominopelvic CT for the first and second year; full colonoscopy after year one if not done preoperatively, then every three years. Additional tests, including a positron emission tomography scan, were performed if needed.

Overall survival (OS) was measured from the surgery date to that of death/last follow-up, whilst disease-free survival (DFS) was measured to that of tumour recurrence. Recurrence was diagnosed through radiological detection of enlarging lesions or histological confirmation. This study followed the STROBE statement for cohort studies [19].

### 2.5. Statistical Analysis

Descriptive statistics are presented with the median and interquartile range (IQR) for quantitative variables. The Chi-square test (or Fisher’s exact test) was used to compare categorical data, and the Mann–Whitney U-test was used to compare non-parametric data. Statistical analysis was performed using IBM SPSS Statistics for Mac, version 28 (IBM Corp., Armonk, NY, USA). Survival and recurrence rates were calculated through the Kaplan–Meier model and compared by a log-rank test. Survival analysis was conducted using the Cox regression model to estimate the Hazard Ratio (HR). Confidence intervals were estimated at 95%, and significance level was set at *p* < 0.05.

## 3. Results

### 3.1. Patient Characteristics

A total of 202 emergency right colectomies were performed with either an open (*n* = 114) or laparoscopic (*n* = 88) approach (Figure 1). The characteristics of patients and primary tumours are listed in Table 1. No differences in age and BMI were identified, but sex was significantly different. The open group had more ASA grade III–IV compared to the laparoscopic group.

### 3.2. Operative Outcomes

Laparoscopic approach indication significantly increased (*p* < 0.001) in the second half of the study period (≥2015). The open group had a higher rate of transverse tumours while the laparoscopic had more ascending and hepatic flexure tumours. More extended right colectomies were performed in the open group (31.6 vs. 18.2%, *p* = 0.031). Conversion occurred in thirteen (14.8%) patients, eight patients for voluminous adherent tumours (pT4, *n* = 5; pT3, *n* = 3), two for adhesions, one for caecal distension with serosal tears, and the data were missing for two cases.

The length of surgery was significantly longer for the laparoscopic group (156 vs. 203 min, *p* < 0.001).

### 3.3. Pathologic Results

Pathological TNM staging did not differ between the two groups. However, lymph node yield was higher in the laparoscopic group. Resection clearance was significantly higher in the laparoscopic group (R0, 78.9 vs. 87.5%, *p* = 0.049), whereas the open group had a higher rate of macroscopically positive resections (R2, 11.4 vs. 2.3%)

### 3.4. Postoperative course

Postoperative stay was significantly shorter in the laparoscopic group (12.5 vs. 8.0 days, *p* < 0.001, Table 2). 

Both complication rates and grade were similar between the two groups. Anastomotic leak, wound infection, and ileus were not different between groups. The open group had a higher reoperation rate (7.0 vs. 4.5%) but was not significantly different. A total of 14 (18.2%) patients had a Clavien–Dindo V with no differences between groups. 

### 3.5. Oncological Prognosis

Adjuvant chemotherapy was administered significantly more regularly in the laparoscopic group (31.6 vs. 50.0, *p* = 0.011). Moreover, the laparoscopic group had a higher rate of adjuvant treatment completion (66.7 vs. 90.9%, *p* = 0.007).

The median length of follow-up was significantly higher in the laparoscopic group (20.5 vs. 33.5 months, *p* < 0.001). The recurrence rate was similar between groups (34.2 vs. 36.4%); however, the open group had a significantly higher death rate during follow up (74.6 vs. 48.9%, *p* < 0.001). Liver (17.5% vs. 17.0%) and peritoneal (15.8 vs. 11.4%) were the most frequent metastatic sites, followed by lung (6.1 vs. 9.1%). Local recurrences (anastomotic and mesocolic) were 3.5% in the open group and 4.5% in the laparoscopic group (*p* = 0.730).

**Figure 2:**
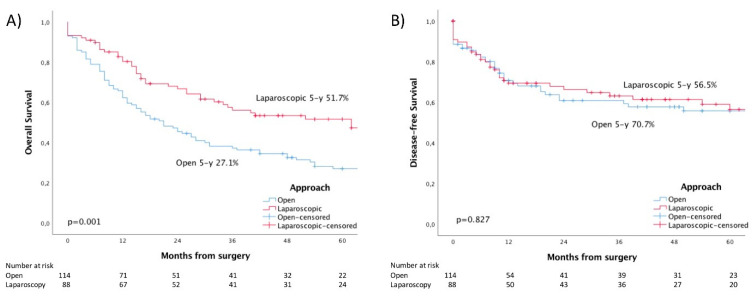
Kaplan–Meier survival curves for (**A**) overall survival and (**B**) disease-free survival.

OS was significantly different (*p* = 0.001) (Figure 2A). OS at one, three, and five years was 62.3, 37.3, and 27.1% for the open group and 80.3, 56.2, and 51.7% for the laparoscopic group. No difference was reported in DFS (*p* = 0.827). DFS at one, three, and five years was 70.7, 60.9, and 55.8% for the open group and 69.4, 63.2, and 56.5% for the laparoscopic group. 

### 3.6. Prognostic Factors

Surgical approach (*p* = 0.002; HR 0.556, 95% CI: 0.385–0.803), pN (*p* < 0.001; HR 2.193, 95% CI: 1.492–3.224), pM (*p* < 0.001; HR 2.679, 95% CI: 1.653–4.343), TNM stage (*p* < 0.001; HR 2.258, 95% CI: 1.531–3.330), retrieved LNs (*p* = 0.035; HR 0.591, 95% CI: 0.362–0.963), R stage (*p* < 0.001; HR 4.529, 95% CI: 2.940–6.975), complication rate (*p* = 0.007; HR 1.619, 95% CI: 1.141–2.296), and complication severity (*p* < 0.001; HR 2.941, 95% CI: 1.928–4.487) were risk factors for OS upon univariate analysis (Table 3). 

Surgical approach (*p* < 0.001; HR 0.449, 95% CI: 0.303–0.666), pN (*p* = 0.005; HR 1.809, 95% CI: 1.194–2.739), pM (*p* = 0.023; HR 1.766, 95% CI: 1.080–2.887), retrieved LNs (*p* = 0.001; HR 0.434, 95% CI: 0.261–0.723), R stage (*p* < 0.001; HR 2.105, 95% CI: 1.369–3.237), and complication severity (*p* < 0.001; HR 3.508, 95% CI: 2.255–5.455) were significant upon multivariate analysis.

Pathological N stage (*p* < 0.001; HR 3.081, 95% CI: 1.757–5.402) and R stage (*p* < 0.001; HR 2.851, 95% CI: 1.595–5.096) were risk factors for OS upon univariate analysis (Table 4). Pathological N stage (*p* = 0.003; HR 2.395, 95% CI: 1.352–4.243) and R stage (*p* = 0.011; HR 2.117, 95% CI: 1.184–3.785) were significant also following multivariate analysis.

## 4. Discussion

This study shows that the laparoscopic approach to emergency right colon cancers can be considered safe in terms of both perioperative and long-term oncological outcomes.

In the current series, emergency laparoscopic right colectomy provided higher lymph node yield, better resection clearance, and shorter length of postoperative hospital stay. The laparoscopic group had higher adjuvant chemotherapy rates with higher completion rates. Five-year OS was significantly higher in the laparoscopic group, while no difference occurred in five years DFS. Surgical approach, pN, pM, retrieved LNs, R stage, and complication severity were significant risk factors for OS upon multivariate analysis. Pathological N stage and R stage were significant risk factors for DFS upon multivariate analysis.

When facing emergency colon cancer, with obstruction, perforation, or bleeding, the principles of oncologic resection should always be aimed for while also considering the medical stability of the patient (i.e., septic status) to obtain a short and uncomplicated postoperative recovery and allow complete oncological staging and adjuvant therapy [3]. In comparison to the older patients, younger patients often present at a more advanced stage, but are more likely to benefit from curative resections [20]. The World Society of Emergency Surgery (WSES) 2017 guidelines on colon cancer emergencies [3] advocate that for stable patients, a right colectomy with primary anastomosis is the preferred option for obstructive cancers, while an end ileostomy with colonic fistula is considered a valid alternative if primary anastomosis is unsafe. For unstable patients, a right colectomy with end ileostomy should be considered the procedure of choice. However, these guidelines do not provide any comment on the surgical approach for emergency right colon cancers. Colorectal societies do not consider the surgical approach when discussing the treatment of emergency right colon cancers. Therefore, the laparoscopic approach for emergency right colon cancers is rarely discussed in the literature [21]. 

Despite laparoscopy being considered the gold standard for elective colorectal oncological resections, it is still generally considered unsafe in the emergency setting due to patients’ general conditions, complicated intra-abdominal diseases, and risk of injury to the distended bowel during manipulation. Nevertheless, the laparoscopic approach to elective right colectomies has shown equivalent or even better short-term outcomes compared to the open approach [15,16]. Moreover, the 2023 National Emergency Laparotomy Audit (NELA) from the UK reported that only 11.1% of audited patients for emergency bowel surgery were operated on laparoscopically, which is significantly less than the percentage discussed in this series. The outcomes presented here, despite potential biases in selection and the limitations of retrospective analyses, suggest that there is a larger patient population that could benefit from minimally invasive emergency surgery, and it is advisable to investigate this further to improve patient care.

Emergency resections are often performed out of hours with staff less experienced in laparoscopic surgery and may not have a specialist colorectal surgeon’s input. Therefore, a potentially long laparoscopic resection is often less desirable in the emergency theatre. The present study confirmed significantly longer surgery for the laparoscopic group (*p* < 0.001), with a median difference of 53 min, which is consistent with a recent meta-analysis [16]. However, our centre had significant experience in laparoscopic colorectal surgery during the Lapco program (2010–2013) [22] and the unit offers a specialist on-call service, hence the “buy-in” for laparoscopic emergency resection was available. 

Laparoscopy in emergency can be associated with a higher conversion rate. The present series showed a conversion rate of 14.8%. However, conversion was performed in all cases not because of injuries or technical difficulties but for oncological safety reasons. The literature lacks data on conversion rates for emergency right colon cancer resections. Ng et al. reported no conversion in a small series of 14 cases [15]. However, the authors reported a series with a lower BMI, age, and pathological staging which could have affected the lower conversion rate. 

The laparoscopic approach provided good oncological outcomes in the present study (number of harvested lymph nodes, curative resections (R0), and OS). The present study showed a higher lymph node yield obtained with the laparoscopic approach (18.0 vs. 22.0, *p* < 0.001), which is a marker for better oncological survival and suggests proper dissection even in an emergency setting. This was in contrast with a recent meta-analysis [10] showing that only one study [23] reported lymph node yield which was not significantly different. Interestingly, in the present series, the open group had a lower rate of lymph node yield even if they had more extended right colectomies. Curiously, despite similar staging between the two groups, the laparoscopic approach provided higher rates of curative resections (R0, 78.9 vs. 87.5, *p* = 0.049).

This study reports equivalence of laparoscopy and open surgery for DFS, and local recurrence. This shows that the surgical approach did not have any effect on recurrence rates and time to recurrence, as previously reported for rectal cancer resection with a laparoscopic vs. robotic approach [24]. However, the laparoscopic group had a higher rate of adjuvant chemotherapy and completion. This could be a result of the better perioperative outcomes, allowing the patients to have a good physical state for undergoing adjuvant treatment. Interestingly, despite no difference in complication grade and severity, patients undergoing the laparoscopic approach survived better. The open approach group had a higher rate of deaths during follow-up (74.6 vs. 48.9%, *p* < 0.001) which could be related more to the patient’s status at presentation than to the surgical approach per se. Unfortunately, due to the retrospective nature of this study, it was not possible to recover the ASA scores of 30.7% of open approach patients. Nevertheless, higher rates of ASA III and IV were reported in the open approach group. The favourable oncological outcomes and mortality in the laparoscopic group could potentially be related to the physiological status of the patients in the perioperative period. Poor physiological status might have precluded laparoscopic intervention as well as adjuvant therapy.

When evaluating the risk factor for OS, the multivariate Cox regression analysis showed that surgical approach, pN, pM, retrieved LNs, R stage, and complication severity were significant, but for DFS, only pN and R stage were significant in the multivariate Cox regression analysis. To the authors’ knowledge, this is the first study evaluating the risk factors for OS and DFS in a series of open and laparoscopic right colectomies in the emergency setting. Although emergency laparoscopic right hemicolectomies have been shown to be feasible in the present study, the authors believe that this approach should be carefully selected preoperatively and re-discussed intraoperatively, as it is not suitable for every emergency case. Small stenotic tumours, with short duration of obstructing symptoms, and mild dilatation of small bowel should be indicated to laparoscopy whereas bulky fixed obstructing tumours, advanced adhesions, and gross bowel distension should not be indicated to laparoscopy for safety reasons. Preoperative abdominal surgery should not be considered as a contraindication to laparoscopic surgery [15].

This study has several limitations. Firstly, it is a retrospective study from a single tertiary care centre potentially suffering from patient selection bias. Secondly, both open and laparoscopic resections were performed by experienced colorectal surgeons in a tertiary high-volume centre; the results may not be generalizable to all institutions. Thirdly, because it is a non-randomized study, the decision to perform an open or laparoscopic operation rested on the surgeon’s preference, which in turn relied on the surgeon’s decision-making ability based on surgical expertise and patient factors. This is likely to introduce a selection bias. Fourthly, the study timeline is quite extended (11 years) with consequent differences in surgical and oncological treatment, pathological evaluation, and perioperative/follow-up protocol. Lastly, this study included all emergency right colectomies (obstruction, bleeding, and perforation), which could be a source of bias for comparison between approaches.

This study has several strengths. It uses a large dataset from a cancer centre with high emergency workload and reports the long-term oncological survival with five-year recurrence rates and location from emergency right colon adenocarcinomas. This study reports the risk factors for OS and DFS for emergency right colon cancers. A prospective randomized study could be useful to confirm and prove the role of the laparoscopic approach in right colon cancers in an emergency setting.

## 5. Conclusions

The laparoscopic approach for emergency right colon cancers provides higher lymph node yield, better resection clearance, and shorter postoperative stay. The laparoscopic group had higher adjuvant chemotherapy rates with a higher completion rate. Five-year OS was significantly higher in the laparoscopic group, while no difference occurred in five years DFS. Surgical approach, pN, pM, retrieved LNs, R stage, and complication severity were significant risk factors for OS upon multivariate analysis. Pathological N stage and R stage were significant risk factors for DFS upon multivariate analysis. However, the results of this study should be interpreted with caution considering its limitations and potential selection biases. The present study suggests that the use of laparoscopic emergency surgery in select patients and appropriate centres is safe and applicable, although prospective randomized controlled studies are needed to confirm these results.

## Figures and Tables

**Table 1:** Series characteristics. Values are presented as number (percentage) or median (interquartile range, IQR). BMI: body mass index; LNs: lymph nodes.

	Total (*n* = 202)	Open (*n* = 114)	Laparoscopic (*n* = 88)	*p*
Age, years	74.0 (63.0–81.0)	76.0 (64.0–81.2)	71.0 (59.5–80.0)	0.137
Male sex (%)	85 (42.1%)	41 (36.0%)	44 (50.0%)	0.045
BMI, Kg/m^2^	24.0 (21.9–28.0)	24.0 (21.3–28.0)	24.0 (22.0–28.0)	0.709
ASA				0.060
I	12 (5.9%)	6 (5.3%)	6 (6.8%)	
II	68 (33.7%)	30 (26.3%)	38 (43.2%)	
III	57 (28.2%)	34 (29.8%)	23 (26.1%)	
IV	11 (5.4%)	9 (7.9%)	2 (2.3%)	
Missing data	54 (26.7%)	35 (30.7%)	19 (21.6%)	
Tumour site				0.012
Caecum	92 (45.5%)	52 (45.6%)	40 (45.5%)	
Ascending colon	35 (17.3%)	17 (14.9%)	18 (20.5%)	
Hepatic flexure	31 (15.3%)	12 (10.5%)	19 (21.6%)	
Transverse colon	44 (21.8%)	33 (28.9%)	11 (12.5%)	
Type of surgery				0.031
Right hemicolectomy	150 (74.3%)	78 (68.4%)	72 (81.8%)	
Extended right hemicolectomy	52 (25.7%)	36 (31.6%)	16 (18.2%)	
Operative time, min	180.0 (136.5–226.0)	156.0 (123.7–211.5)	203 (161.7–242.2)	<0.001
Conversion rate (%)	/	/	13 (14.8%)	/
LN yield, *n*	19.0 (15.0–26.0)	18.0 (13.0–22.0)	22.0 (17.0–30.0)	<0.001
pT				0.339
1	1 (0.5%)	1 (0.9%)	0	
2	8 (4.0%)	5 (4.4%)	3 (3.4%)	
3	81 (40.1%)	40 (35.1%)	41 (46.6%)	
4	112 (55.4%)	68 (59.6%)	44 (50.0%)	
pN				0.341
0	78 (38.6%)	48 (42.1%)	30 (34.1%)	
1	55 (27.2%)	27 (23.7%)	28 (31.8%)	
2	69 (34.2%)	39 (34.2%)	30 (34.1%)	
pM+	22 (10.9%)	14 (12.3%)	8 (9.1%)	0.471
pTNM staging				0.513
1	9 (4.5%)	6 (5.3%)	3 (3.4%)	
2	68 (33.7%)	41 (36.0%)	27 (30.7%)	
3	103 (51.0%)	53 (46.5%)	50 (56.8%)	
4	22 (10.9%)	14 (12.3%)	8 (9.1%)	
R grade				0.049
0	167 (82.7%)	90 (78.9%)	79 (87.5%)	
1	20 (9.9%)	11 (9.6%)	9 (10.2%)	
2	15 (7.4%)	13 (11.4%)	2 (2.3%)	

**Table 2:** Postoperative follow-up and oncological outcomes. Values are presented as number (percentage) or median (interquartile range, IQR). BMI: body mass index; LNs: lymph nodes.

	Total (*n* = 202)	Open (*n* = 114)	Laparoscopic (*n* = 88)	*p*
Postoperative stay, days	10.0 (7.0–18.0)	12.5 (8.0–21.0)	8.0 (5.0–13.7)	<0.001
30d Complication	77 (38.1%)	45 (39.5%)	32 (36.4%)	0.652
Clavien–Dindo				0.454
II	46 (59.7%)	28 (62.2%)	18 (56.2%)	
III	12 (15.6%)	8 (17.8%)	4 (12.5%)	
IV	5 (6.5%)	1 (2.2%)	4 (12.5%)	
V	14 (18.2%)	8 (17.8%)	6 (18.7%)	
Anastomotic leak	4 (2.0%)	2 (4.6%)	2 (2.3%)	1.000
Wound infection	40 (19.8%)	22 (19.3%)	18 (20.5%)	0.838
Ileus	18 (8.9%)	9 (7.9%)	9 (10.2%)	0.564
Reoperation	12 (5.9%)	8 (7.0%)	4 (4.5%)	0.557
Readmission	10 (5.0%)	7 (6.1%)	3 (3.4%)	0.518
Adjuvant chemotherapy, yes	80 (39.6%)	36 (31.6%)	44 (50.0%)	0.011
Adjuvant chemotherapy completion	64 (80.0%)	24 (66.7%)	44 (90.9%)	0.007
Dead in FU	128 (63.4%)	85 (74.6%)	43 (48.9%)	<0.001
Follow-up, months	26.0 (9.0–58.5)	20.5 (7.7–53.2)	33.5 (13.2–62.0)	<0.001
Recurrence	71 (35.1%)	39 (34.2%)	32 (36.4%)	0.751
Liver	35 (17.3%)	20 (17.5%)	15 (17.0%)	0.926
Lung	15 (7.4%)	7 (6.1%)	8 (9.1%)	0.428
Peritoneal	28 (13.9%)	18 (15.8%)	10 (11.4%)	0.367
Uterus/ovaries	3 (1.5%)	3 (2.6%)	0	0.125
PALN	4 (2.0%)	0	4 (4.5%)	0.021
Splenic	2 (1.0%)	1 (0.9%)	1 (1.1%)	0.854
Pelvis	6 (3.0%)	2 (1.8%)	4 (4.5%)	0.247
Anastomotic site/mesentery	8 (4.0%)	4 (3.5%)	4 (4.5%)	0.708
Bone	4 (2.0%)	2 (1.8%)	2 (2.3%)	0.793
Sigmoid	1 (0.5%)	1 (0.9%)	0	0.378

**Table 3:** Univariate and multivariate analysis of overall survival. BMI: body mass index; LNs: lymph nodes.

		Univariate		Multivariate	
		HR (95% CI)	*p*	HR (95% CI)	*p*
Age, years	<55	Reference			
	≥55	1.900 (0.964–3.743)	0.064		
Sex	Female	Reference			
	Male	0.741 (0.518–1.058)	0.099		
BMI, Kg/m^2^	<25	Reference			
	≥25	0.838 (0.550–1.277)	0.411		
Approach	Lap	0.556 (0.385–0.803)	0.002	0.449 (0.303–0.666)	<0.001
pT	0–2	Reference			
	3–4	1.495 (0.611–3.660)	0.379		
pN	0	Reference			
	1–2	2.193 (1.492–3.224)	<0.001	1.809 (1.194–2.739)	0.005
pM		2.679 (1.653–4.343)	<0.001	1.766 (1.080–2.887)	0.023
Retrieved LN	<12	Reference			
	≥12	0.591 (0.362–0.963)	0.035	0.434 (0.261–0.723	0.001
Stage	0–II	Reference			
	III–IV	2.258 (1.531–3.330)	<0.001		
R stage	0	Reference			
	1–2	4.529 (2.940–6.975)	<0.001	2.105 (1.369–3.237)	<0.001
Complication	yes	1.619 (1.141–2.296)	0.007		
Clavien–Dindo	0–II	Reference			
	III–V	2.941 (1.928–4.487)	<0.001	3.508 (2.255–5.455)	<0.001
Adjuvant chemotherapy	yes	0.856 (0.593–1.235)	0.406		

**Table 4:** Univariate and multivariate analysis of disease-free survival. BMI: body mass index; LNs: lymph nodes.

		Univariate		Multivariate	
		HR (95% CI)	*p*	HR (95% CI)	*p*
Age, years	<55	Reference			
	≥55	1.287 (0.590–2.810)	0.526		
Sex	Female	Reference			
	Male	0.709 (0.437–1.150)	0.163		
BMI, Kg/m^2^	<25	Reference			
	≥25	1.205 (0.716–2.028)	0.482		
Approach	Lap	0.950 (0.595–1.517)	0.830		
pT	0–2	Reference			
	3–4	2.064 (0.506–8.423)	0.313		
ypN	0	Reference			
	1–2	3.081 (1.757–5.402)	<0.001	2.395 (1.352–4.243)	0.003
Retrieved LN	<12	Reference			
	≥12	0.837 (0.362–1.936)	0.678		
Stage	0–II	Reference			
	III–IV	1.252 (0.542–2.891)	0.599		
R stage	0	Reference			
	1–2	2.851 (1.595–5.096)	<0.001	2.117 (1.184–3.785)	0.011
Complication	yes	1.348 (0.833–2.183)	0.224		
Clavien–Dindo	0–II	Reference			
	III–V	1.594 (0.809–3.142)	0.178		
Adjuvant chemotherapy	yes	1.570 (0.966–2.550)	0.069		

## Data Availability

All data used was extracted from a prospective research database and already had ethical approval (IRAS ID 293129). The project was registered with the R&D department at Portsmouth Hospitals University NHS Trust. Upon request, anonymised data can be made available as appropriate.

## References

[1] Morgan E., Arnold M., Gini A., Lorenzoni V., Cabasag C.J., Laversanne M., Vignat J., Ferlay J., Murphy N., Bray F. (2023). Global burden of colorectal cancer in 2020 and 2040: Incidence and mortality estimates from GLOBOCAN. Gut.

[2] Abel G.A., Shelton J., Johnson S., Elliss-Brookes L., Lyratzopoulos G. (2015). Cancer-specific variation in emergency presentation by sex, age and deprivation across 27 common and rarer cancers. Br. J. Cancer.

[3] Pisano M., Zorcolo L., Merli C., Cimbanassi S., Poiasina E., Ceresoli M., Agresta F., Allievi N., Bellanova G., Coccolini F. (2018). 2017 WSES guidelines on colon and rectal cancer emergencies: Obstruction and perforation. World J. Emerg. Surg..

[4] Gunnarsson H., Jennische K., Forssell S., Granstrom J., Jestin P., Ekholm A., Olsson L.I. (2014). Heterogeneity of colon cancer patients reported as emergencies. World J. Surg..

[5] McArdle C.S., Hole D.J. (2004). Emergency presentation of colorectal cancer is associated with poor 5-year survival. Br. J. Surg..

[6] Elliss-Brookes L., McPhail S., Ives A., Greenslade M., Shelton J., Hiom S., Richards M. (2012). Routes to diagnosis for cancer—Determining the patient journey using multiple routine data sets. Br. J. Cancer.

[7] Downing A., Aravani A., Macleod U., Oliver S., Finan P.J., Thomas J.D., Quirke P., Wilkinson J.R., Morris E.J. (2013). Early mortality from colorectal cancer in England: A retrospective observational study of the factors associated with death in the first year after diagnosis. Br. J. Cancer.

[8] Renzi C., Lyratzopoulos G., Card T., Chu T.P., Macleod U., Rachet B. (2016). Do colorectal cancer patients diagnosed as an emergency differ from non-emergency patients in their consultation patterns and symptoms? A longitudinal data-linkage study in England. Br. J. Cancer.

[9] Mitchell E.D., Pickwell-Smith B., Macleod U. (2015). Risk factors for emergency presentation with lung and colorectal cancers: A systematic review. BMJ Open.

[10] Cirocchi R., Cesare Campanile F., Di Saverio S., Popivanov G., Carlini L., Pironi D., Tabola R., Vettoretto N. (2017). Laparoscopic versus open colectomy for obstructing right colon cancer: A systematic review and meta-analysis. J. Visc. Surg..

[11] Martinez-Santos C., Lobato R.F., Fradejas J.M., Pinto I., Ortega-Deballon P., Moreno-Azcoita M. (2002). Self-expandable stent before elective surgery vs. emergency surgery for the treatment of malignant colorectal obstructions: Comparison of primary anastomosis and morbidity rates. Dis. Colon. Rectum.

[12] Papageorge C.M., Zhao Q., Foley E.F., Harms B.A., Heise C.P., Carchman E.H., Kennedy G.D. (2016). Short-term outcomes of minimally invasive versus open colectomy for colon cancer. J. Surg. Res..

[13] Athanasiou C.D., Robinson J., Yiasemidou M., Lockwood S., Markides G.A. (2017). Laparoscopic vs open approach for transverse colon cancer. A systematic review and meta-analysis of short and long term outcomes. Int. J. Surg..

[14] Newman C.M., Arnold S.J., Coull D.B., Linn T.Y., Moran B.J., Gudgeon A.M., Cecil T.D. (2012). The majority of colorectal resections require an open approach, even in units with a special interest in laparoscopic surgery. Colorectal. Dis..

[15] Ng S.S., Lee J.F., Yiu R.Y., Li J.C., Leung W.W., Leung K.L. (2008). Emergency laparoscopic-assisted versus open right hemicolectomy for obstructing right-sided colonic carcinoma: A comparative study of short-term clinical outcomes. World J. Surg..

[16] Podda M., Pisanu A., Segalini E., Birindelli A., Pellino G., Marino M.V., Gomes C.A., Kumar J., Di Saverio S. (2020). Emergency right colectomy: Is there a role for minimally invasive surgery?—A sytematic review and meta-analysis of short-term clinical outcomes. Ann. Laparosc. Endosc. Surg..

[17] Weiser M.R. (2018). AJCC 8th Edition: Colorectal Cancer. Ann. Surg. Oncol..

[18] Dindo D., Demartines N., Clavien P.A. (2004). Classification of surgical complications: A new proposal with evaluation in a cohort of 6336 patients and results of a survey. Ann. Surg..

[19] Von Elm E., Altman D.G., Egger M., Pocock S.J., Gotzsche P.C., Vandenbroucke J.P., Initiative S. (2008). The Strengthening the Reporting of Observational Studies in Epidemiology (STROBE) statement: Guidelines for reporting observational studies. J. Clin. Epidemiol..

[20] Costa G., Frezza B., Fransvea P., Massa G., Ferri M., Mercantini P., Balducci G., Buondonno A., Rocca A., Ceccarelli G. (2019). Clinico-pathological Features of Colon Cancer Patients Undergoing Emergency Surgery: A Comparison Between Elderly and Non-elderly Patients. Open Med..

[21] Chand M., Siddiqui M.R., Gupta A., Rasheed S., Tekkis P., Parvaiz A., Mirnezami A.H., Qureshi T. (2014). Systematic review of emergent laparoscopic colorectal surgery for benign and malignant disease. World J. Gastroenterol..

[22] Hanna G.B., Mackenzie H., Miskovic D., Ni M., Wyles S., Aylin P., Parvaiz A., Cecil T., Gudgeon A., Griffith J. (2022). Laparoscopic Colorectal Surgery Outcomes Improved After National Training Program (LAPCO) for Specialists in England. Ann. Surg..

[23] Li Z., Li D., Jie Z., Zhang G., Liu Y. (2015). Comparative Study on Therapeutic Efficacy Between Hand-Assisted Laparoscopic Surgery and Conventional Laparotomy for Acute Obstructive Right-Sided Colon Cancer. J. Laparoendosc. Adv. Surg. Technol. A.

[24] Piozzi G.N., Rusli S.M., Lee T.H., Baek S.J., Kwak J.M., Kim J., Kim S.H. (2022). Robotic approach may be associated with a lower risk of lung metastases compared to laparoscopic approach for mid-low rectal cancer after neoadjuvant chemoradiotherapy: A multivariate analysis on long-term recurrence patterns. Int. J. Colorectal. Dis..

